# Why coding the modality of primary care consultations matters

**DOI:** 10.3399/BJGP.2025.0581

**Published:** 2025-09-23

**Authors:** Stephanie Drew, Benedict Hayhoe, Geva Greenfield, Azeem Majeed, Ara Darzi, Ana Luisa Neves

**Affiliations:** 1 Institute of Global Health Innovation, Imperial College London, London, UK; 2 Academic Department of Military General Practice, Royal Centre for Defence Medicine, Birmingham, UK; 3 Department of Primary Care and Public Health, Imperial College London, London, UK; 4 Department of Primary Care and Public Health, Imperial College London, London, UK; 5 Department of Primary Care and Public Health, Imperial College London, London, UK; 6 Department of Primary Care and Public Health, Imperial College London, London, UK

The UK Government 10-year Health Plan^1^ sets out a strong commitment to deliver on recommendations in Lord Darzi’s recent Independent Investigation of the NHS.^2^ Specifically, it focuses on shifting care from hospital to community, increasing use of digital delivery of care, and disease prevention. In primary care, this can include total triage approaches using online consultations, as well as virtual consultations as a new model for improved patient access and efficiency.

However, evidence on the impacts of virtual consultations on the quality and safety of primary care remains limited. While some qualitative evidence exists, a recent systematic literature review identified only one quantitative, small non-UK based (*n* = 30) study that measured patient safety as an outcome.^3^ One of the factors limiting quantitative research is the inconsistent coding of consultation modality data (for example, face-to-face or virtual) in primary care.^4^

Evidence suggests that national statistics underestimate the volume of virtual consultations. Analysis of data from one of the largest online consultation providers for NHS primary care showed that the number of online consultation submissions alone over one platform in 2021–2023 significantly outnumbered the combined video/online appointment data from NHS Digital,^5^ demonstrating the likely substantial underestimation of appointment volume in national statistics. Evidence suggests that this situation may be continuing. In June 2023, telephone appointments represented 26% of all GP appointments, ‘unknown mode’ appointments accounted for 2%, and video/online appointments made up 1% of all appointments.^6^ For June 2025, the NHS England monthly dataset shows that telephone appointments represented 25% of GP appointments, while 2% (*n* = 703 824) of all appointments continued to be categorised as ‘unknown mode’, and 8% (almost 2.5 million) were categorised as ‘video/online’.^6^

Inaccurate data also lead to a misrepresentation of GP workload and resource requirement. Better data entry would support more efficient resource allocation in primary care and offer a clearer picture of general practice workload, which is key for shaping public understanding and healthcare policy. Improving this situation would align with recent calls for practice in primary care to be guided by real-world, primary care-specific research.^7^

## Why is consultation modality poorly recorded?

Primary care staff tend to code more accurately when the clinical codes are clearly linked to patient care outcomes or when there are financial incentives involved.^8^ However, codes that contribute to the achievement of targets, such as the Quality and Outcomes Framework, and therefore funding, are not always the same as those that facilitate research. Consultation modality coding does not currently contribute towards funding, nor is it likely to be seen by primary care staff as something that is critical to patient care.

There are many electronic GP medical record systems used in the UK, each with different ways of documenting appointment modality.^6^ Although appointment type is set locally by individual practices, NHS Digital has noted that the data recorded may not accurately reflect the nature of the consultation:^6^ for example, virtual consultations delivered by telephone or video may be logged as face-to-face; telephone triage sessions may be recorded as a single block rather than discrete consultations, thereby underestimating activity; and modality labels can be imprecise, with ‘online/video’ appointments not necessarily involving any video components.^6^

Moreover, effective coding requires sufficient training, which has been recognised as a potential factor contributing to inconsistent clinical coding, especially among clinical staff.^8^ To improve the accurate documentation of clinical data, SNOMED CT is used. Guidance from NHS England indicates several benefits of using SNOMED CT, including improved data analysis to support clinical research. Primary care providers have had this function enabled since 2018.^9^ SNOMED CT codes can indeed be used for modality; however, there is an issue with duplicate codes. Multiple codes are available that purportedly indicate a telephone consultation; for example, ‘consultation by telephone’, ‘telepractice consultation’, ‘remote verbal consultation’, and ‘telephone consultation’. In other cases, such as ‘assessment via multimedia encounter type’, it is not clear whether it indicates a video consultation or other type of modality.^4^ This exemplifies a previously identified barrier to coding — clinicians not being able to identify the correct code and free texting instead due to time pressures.^8^

## Improving consultation modality coding

It is important for practices and their staff, GP system suppliers, and researchers to note these difficulties and work towards improving the situation. There are several promising avenues for improvement.

At national level, aligning coding of modality and appointment booking practices across medical centres would increase consistency in data quality. Coding should be straightforward and easily accessible. Reducing duplicate codes available to clinicians would assist in improved data quality in the face of time pressures. Introducing financial incentives for accurate modality coding, and indeed for any other codes used in research not already aligned to clinical targets, would improve primary care-based research capabilities.

At practice level, staff should be encouraged to follow guidance on consultation modality coding. The importance of accurate coding for research must be highlighted alongside the importance of coding for clinical purposes. Templates are known to improve data entry by primary care staff^8^ and could be adapted to include an indication about the consultation modality. Practices should endeavour to code and capture their workflow practices and appointment data accurately, ensuring that each appointment is separately recorded.

Digital scribes may also improve data entry, reducing the burden of documentation for clinicians.^10^ Digital scribes, including ambient voice to text artificial intelligence systems, stand to improve data entry for clinical, research, and workload recognition purposes.^11^ They will need to use SNOMED CT clinical coding to do so and their accuracy in performing this task will be essential to avoid clinical error, ensure data robustness, and be interoperable with existing systems. If this can be achieved, it would confer significant benefits to clinician workload and research capabilities.

Consultation modality coding could be otherwise automated by integrating software systems such that using video or telephone software results in the auto-coding of modality into the electronic health record, improving data quality and removing the burden of coding from busy clinicians. An integrated system with single sign on and a single patient record will facilitate this. Mechanisms to increase the robustness of modality data, accommodating last minute changes to consultation mode, should be introduced.

## The future of data-driven primary care

As virtual consultations are increasingly adopted in primary care, there remains limited understanding of their impact on quality and safety. Without accurate coding of consultation modalities, we lack the data needed to evaluate the effects of introducing virtual consultations. Ensuring precise and consistent data entry is therefore critical to generating evidence on new approaches to care. Coordinated efforts to optimise coding would enable quantitative, data-driven insights into virtual patient care, ensuring that the volume and nature of care provision are accurately captured, and supporting the safe evolution of primary care within the NHS as it transitions from analogue to digital.
